# HOME CARE EXECUTIVES SAY MEDICARE HOME HEALTH PAYMENT SYSTEM ENCOURAGES LESS-IS-BETTER PRACTICE

**DOI:** 10.1093/geroni/igz038.1104

**Published:** 2019-11-08

**Authors:** William D Cabin

**Affiliations:** Temple University, Philadelphia, Pennsylvania, United States

## Abstract

There has been an increasing trend for Congress and the Centers for Medicare and Medicaid Services (CMS) to add non-skilled services to coverage under Medicare Advantage and Medicaid inpatient hospital. At the same time there has been a 75% decline in home health aide visits, the only Medicare home health non-skilled service, as a percentage of all Medicare home health visits from 2000-2016. A literature review indicates no studies addressing the potential factors accounting from these seemingly contradictory trends. The present study is based on interviews of five Chief Executive Officers (CEOs), five Chief Financial Officers (CFOs), and eight Chief Nursing Officers (CNOs) from Medicare-certified home health agencies between October 2017-July 2018. Results indicated agreement among interviewees on three themes: the Medicare home health relies on a medical model which focuses on intermittent skilled care; the Medicare home health prospective payment system (PPS) exacerbated the focus on skilled care by rewarding higher reimbursement for skilled care based episodes; and a synergy has evolved of “less is better” regarding utilization of home health aide services and reimbursement. Policymakers are urged to consider adding coverage of non-skilled services under Medicare home health, similar to Medicare Advantage, by funding demonstration projects with appropriate changes in reimbursement.

